# Maximal and Explosive Strength of High-Level Alpine Skiers After Severe Lower Extremity Injury: A Retrospective Comparison with Non-Injured Skiers

**DOI:** 10.3390/sports13120450

**Published:** 2025-12-11

**Authors:** Simon Trachsel, Micah Gross, Björn Bruhin, Heiner Baur, Klaus Hübner

**Affiliations:** 1 Department of Elite Sport, Federal Office of Sport, 2532 Magglingen, Switzerland; micah.gross@baspo.admin.ch (M.G.); bjoern.bruhin@swiss-ski.ch (B.B.); klaus.huebner@baspo.admin.ch (K.H.); 2 School of Health Professions, Division of Physiotherapy, Bern University of Applied Science, 3007 Bern, Switzerland; heiner.baur@bfh.ch; 3 Swiss-Ski School, Department of Research and Development, 3048 Worblaufen, Switzerland

**Keywords:** alpine skiing, severe injury, return to sport, maximal strength, explosive strength

## Abstract

Before returning to sport (RTS) following lower extremity injury, competitive alpine skiers’ performance strength profiles should be verified. This study examined whether differences in maximal isometric (F_max_) and explosive strength (P_max_) exist between non-injured (n_INJ) and post-injured (p_INJ) elite skiers (n = 56) after RTS. It also explored whether F_max_ and P_max_ values recover differently over time and whether restoration rates differ between males and females. An explorative analysis was conducted to determine differences in back-squat F_max_ and P_max_ during squat (SJ) and countermovement jumps (CMJ) without and with additional load. Data were available from before injury and after athletes’ RTS for p_INJ or twice across a comparable time span for n_INJ. While differences between n_INJ and p_INJ after rehabilitation are not significant for Fmax, p_INJ generally display significantly lower Pmax (r = 0.34–0.40). Additionally, results suggest that F_max_ is restored first, followed by P_max_ without eccentric component (SJ), and that P_max_ (CMJ) with eccentric component is restored slowest. Further, p_INJ showed lower P_max_ in loaded jumps even before injury (*p* = 0.035–0.047, r = 0.36–0.39). Finally, females display generally lower P_max_ for a given F_max_. Overall, these results contribute to improving rehabilitation and prevention.

## 1. Introduction

Alpine ski racers are exposed to an increased risk of injury while participating in their sport. This can be explained by the characteristics of the sport, such as the high speed, enormous forces acting on the body, and external influences such as material or competition conditions [1,2,3]. The injury incidence is high and has been documented as being more frequent in speed disciplines such as Downhill and Super-G than in technical disciplines such as Giant Slalom and Slalom [2]. The proportion of severe injuries is 38.3 per 100 athletes per season [4]. Sixty-eight percent of all injuries affect the lower extremity (below the waist) [5]. While injuries to the knee, groin/hip, lower leg, ankle and foot are all common, the most frequently diagnosed injury in alpine ski racers is anterior cruciate ligament (ACL) rupture [6].

Successful return to sport (RTS) in competitive athletes following injuries such as ACL ruptures, as well as the rehabilitation process, has often been reported in the literature. The RTS rates in competitive athletes following ACL rupture (46–90%) [7,8,9] are reported to be more widely distributed than for other severe lower extremity injuries (74–89%) [10,11,12]. The specific RTS rates are not clearly documented for any of the severe lower extremity injuries in alpine skiers. However, there are examples of skiers who have returned to the same or even higher level of performance after ACL ruptures [13]. It is worth noting that the re-injury rate for alpine skiers after an ACL injury is high at 47% [14].

A continuous physical and psychological build-up during rehabilitation after a severe injury aims to restore resilience and ensure that athletes can return safely and sustainably to their sport. Athletes and interprofessional rehabilitation teams should have valid criteria for deciding if and when athletes are ready to return to their sport [15,16]. The current literature recommends five categories of RTS criteria: psychological factors, performance/functional testing, strength testing, evaluation of modifiable and non-modifiable risk factors, and time [17]. Return to performance (RTP) represents a further step beyond RTS and entails restoring or improving the athlete’s sport-specific performance factors [18,19]. Whereas RTS assessment protocols described in the literature vary in several aspects, there is a general consensus that tests for sport-relevant strength factors are essential for supporting RTS and RTP decision-making [20]. Consequently, this project will focus exclusively on various specific strength factors assessed during the RTS process.

Two fundamental physical characteristics of alpine skiers are high lower-body maximal and explosive strength, which are required to precisely modulate high muscle force while turning, jumping and landing. Although the relative importance of maximal and explosive strength vary across alpine skiing disciplines, the existing literature suggests a positive correlation between these strength factors and performance in alpine ski racing [21,22]. Specific measurements that have been reported as indicators of these maximal and explosive strength abilities are peak isometric force (F_max_) in the squat, or peak mechanical power (P_max_) in vertical jumps such as the countermovement jump (CMJ) and the squat jump (SJ), respectively.

In day-to-day work with athletes undergoing rehabilitation on their way to RTS, several subjective observations have already been made concerning strength and power deficits. For example, restoring explosive strength seems to be more challenging following severe injury than restoring maximal strength. However, to provide sound advice and guidance to athletes returning to sport after a severe injury, it is essential to better understand the potential differences in the restoration of various strength qualities following such an injury. Therefore, this study aims to provide objective data to support or refute subjective observations and thus improve the criterion-based decision-making process for RTS or RTP. Specifically, maximal and explosive strength characteristics are compared between post-rehabilitation and non-injured skiers.

Thus, the main aim of this study was to test the hypothesis that maximal and explosive strength in alpine skiers upon returning to sport from a severe lower extremity injury differ from those of their non-injured counterparts. Additional aims were to test the hypotheses that (1) maximal and explosive strength values improve at different rates during rehabilitation and (2) that restoration rates of maximal and explosive strength characteristics differ between males and females.

## 2. Methods

### 2.1. Study Design

To address the research questions, this study employed an explorative analysis of routine performance test data [23], combined with a retrospective questionnaire on injury history. For athletes who had suffered severe lower-limb injury and returned to sport thereafter, performance test results from prior to injury and following return to sport were compared with test results from non-injured counterparts at analogous time points. The study was conducted in accordance with the Declaration of Helsinki, and approved by the Ethics Committee of the Canton of Bern, Switzerland (protocol code 2018-00742).

### 2.2. Participant Characteristics

High-level athletes were recruited during routine (federation-endorsed) performance testing in the spring or fall of 2023. Thus, to be included in the study, athletes must have been selected to the Swiss national ski team for the 2023/24 season. This selection entailed having competed at World Cup and European Cup competitions or national FIS (International Ski Federation) races. Further, only athletes who had been in the national team selection pool for at least one prior season were eligible, since first-year athletes would not have had the necessary performance test history to be included in the analysis (see Section 2.4). This study only included athletes without severe lower extremity injuries, or who had recovered from them. Those with a history of other severe injuries (e.g., back injuries) were excluded. A total of 56 complete data sets from alpine skiers aged between 17 and 30 were available for analysis. These included performance test data and injury history information stemming from the years 2018–2023. Descriptive characteristics of the athletes are presented in Table 1.

### 2.3. Data Collection

According to the ski federation’s performance concept, routine performance tests of maxi-mal and explosive strength (details in Section 2.4 below) are carried out twice a year: at the beginning of the preparation period (between late April and early June) and shortly before the competitive season (in late September or October). While performance tests were mandated for some training groups, participation in the study was entirely voluntary. Informed consent was obtained from all subjects involved in the study. Athletes who consented to participate in the study completed an injury-history questionnaire using a secure online questionnaire service (LimeSurvey Cloud, version 5.6.8, Hamburg, Germany) during one of their visits to the performance testing facility in 2023. The questionnaire was created specifically for the investigation to collect injury information corresponding to the time of the performance test data and a period of two years prior. In this manner, it was assured that all included athletes had been free of severe lower-limb injury for at least two years prior to their first included test result. The questionnaire was available in athletes’ native language, was an extension of the Swiss Olympic Federation’s preparation checklist for routine performance testing [24], and based on the injury surveillance system (ISS) used by FIS [25]. Furthermore, it aligned with the current recommendation for injury assessment in competitive sports [26]. Data on injured body region, type of injury, date of injury, and duration of rehabilitation were central themes in the questionnaire. Athletes were informed ahead of time about the study and the main questionnaire items in order to ensure a reliable response.

### 2.4. Performance Test Measurements

Performance testing was based on a validated procedure [23,24]. This consisted of a standardized warm-up and standardized assessments of maximal and explosive lower-limb strength using force plates (MLD Test EVO2 with accompanying software Cyccess, CYC 2.7, SP Sport, Innsbruck, Austria).

First, peak isometric back squat force (F_max_), as a measure of maximal strength, was assessed bilaterally with knee angles of 70° (F_max_ _70) and 100° (F_max_ _100). Note that the knee angles correspond to the biomechanical measuring method. Two trials were allowed for each knee angle. Athletes were instructed to increase force to the ground continually over 2–4 s. Recording frequency was 1000 Hz. Trials with obvious spikes in the force-time signal were deleted and repeated the peak force over 1 ms was retained from the remaining trials. Peak force values were expressed relative to body mass in N/kg.

**Figure 1:**
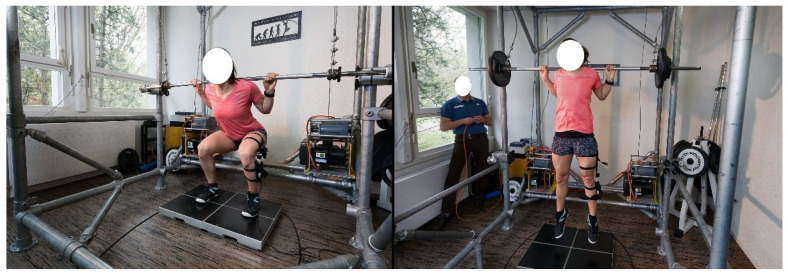
Isometric maximal strength and explosive strength test setting. **Left**: measurement of the bilateral isometric squat with 70° knee flexion on a force plate, with a goniometer for angle control and a fixed bar. **Right**: explosive vertical jump test with additional load. In the background on the floor is the restraint system that catches the bar after the jump.

Next, as a measure of explosive strength, peak mechanical power (P_max_) was measured during countermovement jumps (CMJ) and squat jumps (SJ), each performed without and with an additional load equal to 100% body mass. Jumps without additional load were performed with hands placed on the hips. Additional loads were in the form of a barbell placed upon the shoulders as in the back squat. A retention system (Figure 1) ensured that athletes did not have to land with the barbell on their shoulders. Squat jumps were performed from a starting knee angle of 95–100° (controlled with an electronic goniometer or in some cases visually). Whereas CMJ depth was not standardized, athletes were instructed to descend slightly less than for the SJ and the software provided feedback after each jump in order to ensure this was the case. For each jump condition, multiple trials were allowed to ensure proper execution and verification of athletes’ maximal ability. From the force-time signal, mechanical power was calculated within the software. For the analysis, P_max_ values were retained for each of four jumping conditions. All P_max_ values were expressed relative to body mass in W/kg and reported with the corresponding jump type and load (for example, P_max__CMJ_0 for maximal power of CMJ with no additional load).

The coefficients of variation of the described F_max_ and P_max_ measurements are 2.5–4.7% [23].

### 2.5. Data Analysis

Based on injury history questionnaires, participants were divided into two groups. Those who had suffered a severe lower extremity injury, i.e., an injury that disrupted training for at least 28 days [26] and at most 365 days, were assigned to the post-injured (p_INJ) group. Those who had not suffered severe injury (to any part of the body) were assigned to the non-injured (n_INJ) group.

For each athlete in p_INJ, results from the last performance test conducted prior to injury (T1) and the first test conducted after RTS (T2) were analyzed. RTS was defined as when an athlete was fully reintegrated into the competitive sports system and able to participate in specific training sessions or competitions, depending on the season. In most cases, injured athletes missed one routine test period and thus T1 and T2 were at the same time of year and approximately one year apart. For the n_INJ group, data from two test time points separated by an analogous time interval were analyzed.

Injuries of p_INJ were categorized by type of damage and affected tissue and cases in each category were counted. Descriptive statistics for the duration of disrupted training due to injury, the time span from injury to T2 and from RTS to T2, as well as performance test parameters were calculated for each group and for both sexes with each group. For performance test data, a Shapiro–Wilk test was used to test for normal distribution. A Mann–Whitney U test was used to assess group differences between n_INJ and p_INJ at time points T1 and T2 and to assess sex differences between the n_INJ and p_INJ groups at these time points. The alpha level was set at 0.05. The effect size as a biserial correlation (r) was de-fined classified as large (>0.5), moderate (>0.3) or small (<0.3) [27]. All statistical analyses were performed using JASP software (version 0.17.2.1, Amsterdam, The Netherlands).

## 3. Results

### 3.1. Injury Types and Time Spans

The injury survey identified 32 datasets from non-injured athletes, including 18 males and 14 females, and 24 datasets (seven males and 17 females) from athletes who had suffered a severe lower-limb injury within the relevant period. The most commonly affected body part was the knee (n = 16), followed by the ankle (n = 5) and lower leg (n = 3). Joint sprains and ligament tears (n = 16) were the most frequently reported injuries, followed by fractures (n = 5), bone contusions (n = 2), and cartilage injuries (n = 1). The ACL rupture, in combination with other concomitant knee injuries, was the most common injury (n = 9). In the n_INJ group, the mean time from T1 to T2 was 369 days (SD = 20.1, min. 322, max. 416). In the p_INJ group, the mean time from T1 to T2 was 368 days (SD = 33.1, min. 279, max. 422). The average duration from injury occurance until RTS in p_INJ was 155 days (SD = 73.3, min. 45, max. 260). The mean time from RTS to T2 was 69.5 days (SD = 72.2, min. 0, max. 233). Thus, p_INJ athletes completed T2 on average 225 days (SD = 55.8, min. 109, max. 354) after the occurrence of injury.

### 3.2. Comparison of the Non-Injured and Post-Injured Groups

Descriptive data at T1 and T2 and statistical results for these parameters are listed in Table 2 and Table 3, respectively. It should be noted that the different number of values for the measurements in Table 2 is due to the federation testing practices (first-year athletes do not perform jump tests with an additional load of 100% body weight) and, in some cases, to medical restrictions. At T1 and T2, group means for all F_max_ and P_max_ parameters were lower in p_INJ than in n_INJ. At T1, the two groups differed significantly and with moderate effect sizes for F_max__70 (*p* = 0.022, r = 0.36), CMJ_P_max__100 (*p* = 0.035, r = 0.39), and SJ_P_max__100 (*p* = 0.047, r = 0.36). At T2, differences in SJ_P_max__100, CMJ_P_max__0 and CMJ_P_max__100 reached statistical significance with moderate effect sizes (*p* = 0.017–0.038, r = 0.35–0.38).

### 3.3. Sex Differences Within the Non-Injured and the Post-Injured Groups

The descriptive data and statistical differences between the groups in test situations T1 and T2, separated by sex, are presented in Table 4 and Table 5. In all comparisons, regardless of group or test time point, the females had lower mean values than males, and several of these differences reached significance. In addition to body mass (*p* = <0.001, r = 0.89–0.97), relative maximal strength in the deeper position (F_max__70) was significantly lower in females (*p* = 0.001–0.034, r = 0.44–0.80), and the effect was more pronounced for p_INJ. In contrast, relative maximal strength measurements in the higher position (F_max__100) showed no significant sex-related differences for either group or time point.

Regarding explosive strength (P_max_), females had generally lower values than males. In the n_INJ group, females’ values for most P_max_ parameters and time points were significantly lower than males’, with a large effect size (*p* = <0.001–0.01, r = 0.61–0.81), the only exception being SJ_P_max__100 at T1. In the p_INJ group, females displayed significantly lower values in all P_max_ parameters except CMJ_P_max__0 at T1 (*p* = 0.002–007, r = 0.71–0.82).

## 4. Discussion

The present study aimed to compare the maximal and explosive strength of alpine skiers before and after severe lower extremity injury to those of non-injured skiers. This explorative analysis showed that alpine skiers had slightly lower maximal leg strength (F_max_) values upon returning to competition following a severe lower extremity injury than before injury, but not significantly less compared to their non-injured counterparts. This result is not surprising since modern guidelines recommend the restoration of neuromuscular performance as an elementary factor in rehabilitation in competitive sports [17]. However, this finding contrasts with other studies in which strength deficits in isolated single-joint movements were measured on an isokinetic device up to two years postoperatively after ACL injury [16,28]. Due to the lack of comparative studies between isokinetic single-joint and isometric multi-joint strength measurement methods and the fact that severe injuries to any part of the lower extremity were included in the present study, no further conclusions can be drawn. According to Cross and colleagues, a high maximal leg strength, which they assessed with an isometric mid-thigh pull, is an essential physical ability for performance in alpine skiers, especially in the speed disciplines [21]. The current study used an isometric squat measurement that was already familiar to the athletes. Although the isometric squat is arguably just as valid as the mid-thigh pull [29], F_max_ values are not directly comparable because the knee and hip angles and the bar position are different. Given that no significant differences in F_max_ were observed between n_INJ and p_INJ groups at T2, it can be concluded that athletes had regained the required maximal strength level following rehabilitation from their severe lower extremity injuries at that time point.

On the other hand, significantly lower P_max_ values of p_INJ compared to n_INJ at T2 indicate that explosive strength deficits persisted after skiers had returned to competition following a severe lower extremity injury. The deficits, seen for both loading conditions in the CMJ and in the SJ with additional load, were practically speaking quite large (8.5–10.0%). These results are consistent with a Canadian study on female alpine skiers, which showed differences in unloaded CMJ performance up to five years after ACL injury [30]. Although ACL injuries were not the only injuries included in that study, the observed deficits we found are not surprising given the shorter time period between the injury and the T2 in the current study (mean = 225 days, SD = 55.8). Unfortunately, there are no comparative data in the literature on loaded jumps in relation to RTS in high-level athletes. The lack of significant difference between groups in the SJ power without additional load at T2, while significant deficits with additional load and in CMJ P_max_ at both loading conditions remained, indicates that concentric-only power may return to pre-injury levels sooner during the rehabilitation process than the ability to produce power within a stretch-shortening cycle. This seems plausible since SJ power is generally more influenced by maximal strength (which was restored completely in p_INJ). CMJ power, on the other hand, is more demanding in terms of neuromuscular coordination and eccentric strength. These abilities may therefore be the ones that recover more slowly following severe lower extremity injury in skiers.

Overall, comparisons between n_INJ and p_INJ groups suggest that skiers’ strength abilities recover in the following order during rehabilitation from a severe lower extremity injury:

1. Maximal strength (F_max_)
2. Concentric explosive strength (P_max_ in the SJ)
3. Stretch-shortening explosive strength (P_max_ in the CMJ)

Since our findings are purely observational, it remains to be differentiated between the body’s natural restoration processes and the influence of the rehabilitation intervention. It is feasible that the order of restoration we found is due to the probability that the athletes in the p_INJ group had undergone a rehabilitation program that was initially mostly strength-oriented, and where jumps, and even more so plyometrics, were added toward the end. In any case, this differentiation between strength abilities would have gone undetected without the employment of unloaded and loaded jumps. Current literature describes the need to establish objective, functional, and sport-specific maximal and explosive strength criteria for safe RTS and RTP [31]. The values reported here for F_max_ (particularly F_max__70) and P_max_ fulfill this requirement, especially considering their validity as a performance indicator in alpine skiers and the well-established reference values for this group of athletes. Further studies are needed to help solidify the performance-critical limits of these parameters for the RTS process in alpine skiers; however, the present data can be used as valuable reference values in the criterion-based decision-making process for RTS. As these parameters are currently valid for alpine skiers, a further step would be to extend the research to other sports with a high proportion of maximal and explosive strength.

Another unexpected finding in the current study was the significant differences between n_INJ and p_INJ groups at T1 (prior to injury) in the p_INJ group. While F_max__70 was slightly lower (4.1%) for p_INJ at T1, more meaningful differences (around 8%) were found in P_max_ with additional load in both SJ and CMJ. These differences, measured during routine performance tests, suggest that athletes with poorer explosive strength, especially measured against higher loads (100% of body weight in the current study), may be more prone to severe lower extremity injuries than athletes with greater explosive strength. If this is the case, a minimum required P_max_ level could be identified and applied for interpreting performance tests and giving strength training recommendations. Other studies have frequently described unloaded CMJ and SJ measurements either as routine performance tests or as RTS tests. However, the greatest differences in P_max_ at T1 in the current study were evident in the jumps performed with an additional load; this could indicate an advantage to testing with additional loads not only for performance, but also for prevention and rehabilitation. In complex competitive sports, susceptibility to injury is multifactorial and it is difficult to isolate intrinsic risk factors such as strength. Further prospective research is needed on existing recommendations to more accurately predict injury susceptibility [32].

Using the maximal and explosive strength tests presented here to track athletes’ rehabilitation progress, we found that males and females restored their relative strength abilities to a similar degree and at a similar rate following injury. However, it is important to recognize that females typically display lower relative explosive strength for a given relative maximal strength level than males. This is shown by the sex comparisons at T2, where differences of 8.4–13.6% were documented for F_max__70 compared to differences of 15.1–19.7% for P_max_ in the various jumps. This effect is also described in the literature and is explained by the different muscle structure of male and female athletes in sports where maximal and explosive strength limit performance [33] The differences between the individual abilities and the characteristics of the sex-specific performance profile should therefore be taken into account in performance diagnostics and RTS decision making.

The injury data collection was based on the FIS ISS [25]. This form is well established in alpine ski racing, as it is used for all injuries occurring during official FIS training sessions and competitions, even retrospectively. As our retrospective survey was conducted over a longer period than in comparative studies, measures to reduce recall bias were proactively planned and implemented, as described in the Methods section. Furthermore, it can be assumed that athletes remember incisive events such as serious injuries during their career. In any case, no athlete complained about difficulties in completing the questionnaire.

**Figure 2:**
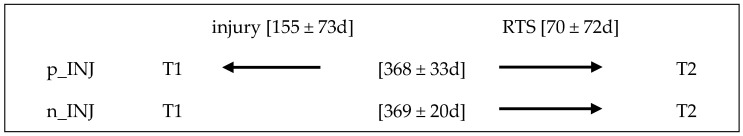
P_INJ: group of athletes who had been severely injured. N_INJ: control group of non-injured athletes. For p_INJ, T1 and T2 performance tests results were defined as those from the last routine performance test prior to injury and the first routine performance test after returning to sport, respectively. For n_INJ, T1 and T2 results were taken from two routine performance tests separated by approximately the same time span as in p_INJ.

Data from routine performance tests, which are carried out twice a year, were used to assess power performance factors. As shown in Figure 2, players returning from serious injuries are recorded during a period of time after their return. Despite the delayed testing, the described deficits are still present, which further supports the T2 comparison results. The varying time span also indicates that RTS testing with elements analogous to the performance tests described should be offered to provide more targeted support for athletes in the final rehabilitation phase and monitor progress. It should be noted that, while ACL injuries are the most commonly documented in alpine ski racing, this study included all severe lower-extremity injuries, as the focus was on performance after returning from a severe injury rather than on the injury itself. Whereas athletes generally do not return to competitive sport for approximately nine months after ACL ruptures [16]., completion of rehabilitation and RTS for the cases included in this study occurred within 155 days on average. This shorter total time span from injury to RTS in the current study was influenced by non-ACL injury cases, which required less time for rehabilitation.

### Limitation

As is commonly the case for studies in high-level athletes, the number of subjects is a limiting factor in statistical analysis. However, our sample size was very appropriate for a study of this nature. The restrictions imposed during the COVID-19 pandemic and the testing practices adopted by the ski federation also contributed to a reduction in the number of participants in this study. Many athletes were not eligible for the study because their performance test history was too sparse or because of injuries that did not affect the lower extremity. Further, because performance testing was mandated by the federation for younger, less-experienced skiers but optional for more-experienced skiers, there was a slight bias in the available data away from veteran athletes. The population described consisted mainly of middle-aged and power athletes. In future studies, it would be ideal to have a balanced distribution between sexes and performance levels (FIS, EC, WC). The continuation of the data collection will increase the number of participants, lead to more precise results, and allow for more accurate statistical methods. Longitudinal development with several years of follow-up using the comprehensive or comparable test procedure with sport-specific strength parameters can further develop the RTS decision factors.

## 5. Conclusions

Due to the high rate of injury in alpine ski racing, with many injuries affecting the lower extremities, it is important to ensure athletes’ readiness when returning to sport (RTS). This study identifies significant differences in performance-relevant strength parameters between non-injured and post-injured athletes after returning from severe lower extremity injuries in alpine ski racing.

In summary, maximum strength is the first strength parameter to return to pre-injury levels and was, for all practical purposes, restored upon RTS in the studies cases. Explosive strength, especially when it entails an eccentric component, takes more time to recover, and this ability was not completely restored in athletes who had been cleared to return to sport. After returning from a severe injury, the differences in relative F_max_ and P_max_ between males and females are similar to those seen before and are also similar in post-injured and non-injured athletes. Finally, it was observed that athletes who would later suffered a severe lower limb injury had a lower maximal isometric force in deep squat position and a lower explosive force in weighted jumps before their injury, compared to those who did not suffer this type of injury. This observation warrants further investigation.

These findings have implications for improving RTS or RTP rehabilitation and criteria-based decision making, as well as injury prevention in athletes.

## Figures and Tables

**Table 1:** Participant characteristics overall and by sex.

	Total (n = 56)		Females (n = 31)		Males (n = 25)	
Age [years]	22.3	±3.0	22.1	±3.5	22.5	±2.5
Height [cm]	172.7	±8.4	166.8	±4.3	180.0	±6.0
Body Mass [kg]	75.1	±10.9	67.3	±6.8	84.7	±6.1

Participant characteristics are reported as mean ± standard deviation with the number of cases (n), for the entire included cohort (total) and by sex.

**Table 2:** Descriptive data of the non-injured and post-injured groups in T1 and T2.

Parameter	Group	n	T1 Mean	SD	n	T2 Mean	SD
F_max__70	n_INJ	32	26.2	±2.3	32	26.4	±2.2
	p_INJ	24	25.1	±2.5	23	25.4	±2.7
F_max__100	n_INJ	29	37.5	±4.4	32	37.7	±4.7
	p_INJ	21	36.4	±4.1	22	35.7	±3.6
CMJ_P_max__0	n_INJ	32	58.3	±8.5	31	58.8	±10.2
	p_INJ	23	54.8	±7.3	23	52.9	±8.2
CMJ_P_max__100	n_INJ	26	52.3	±6.2	29	51.7	±7.6
	p_INJ	17	48.2	±7.4	21	47.3	±6.5
SJ_P_max__0	n_INJ	32	54.0	±7.2	31	53.8	±9.3
	p_INJ	23	51.7	±6.7	23	50.7	±7.8
SJ_P_max__100	n_INJ	26	49.1	±6.1	28	48.1	±8.0
	p_INJ	17	45.3	±6.9	21	43.1	±6.4

Descriptive data for the non-injured (n_INJ) and the post-injured (p_INJ) groups are given for each measurement parameter with the number (n), mean, and standard deviation (SD). Maximal strength parameters (F_max_) are given in newtons per kilogram of body mass (N/kg). Explosive strength parameters (P_max_) for countermovement jump (CMJ) and squat jump (SJ) without (_0) or with an additional load of 100% body weight (_100) are given in watts per kilogram of body mass (W/kg).

**Table 3:** Comparisons between non-injured and post-injury groups by test time point.

Parameter	T1			T2		
	*p*		r	*p*		r
F_max__70	0.022	*	0.36	0.072		0.29
F_max__100	0.435		0.13	0.191		0.21
CMJ_P_max__0	0.093		0.27	0.017	*	0.38
CMJ_P_max__100	0.035	*	0.39	0.038	*	0.35
SJ_P_max__0	0.155		0.23	0.210		0.20
SJ_P_max__100	0.047	*	0.36	0.023	*	0.38

The statistical results of the group comparison of non-injured (n_INJ) and post-injured (p_INJ) athletes are presented for each measured parameter and test time point. The significant differences, indicated by an asterisk and a value of *p* < 0.05, are highlighted and accompanied by the effect size (r).

**Table 4:** Sex-specific descriptive data of the non-injured and post-injured groups at T1 and T2.

Parameter	Sex	N_INJ						P_INJ					
		T1			T2			T1			T2		
		n	Mean	SD	n	Mean	SD	n	Mean	SD	n	Mean	SD
Body Mass	f	14	65.7	±7.2	14	66.9	±7.6	17	67.2	±7.3	17	67.7	±6.4
	m	18	83.7	±7.2	18	84.3	±6.4	7	87.0	±4.8	7	85.8	±5.8
F_max__70	f	14	25.0	±2.7	14	25.1	±2.0	17	24.2	±1.8	16	24.2	±1.9
	m	18	27.1	±1.5	18	27.4	±1.8	7	27.4	±2.5	7	28.0	±2.3
F_max__100	f	12	37.3	±5.5	14	36.1	±3.9	16	35.7	±3.4	16	35.0	±3.8
	m	17	37.6	±3.6	18	38.9	±5.0	5	38.9	±5.3	6	37.6	±2.3
CMJ_P_max__0	f	14	52.7	±6.8	13	51.5	±6.4	16	52.5	±4.8	16	49.6	±6.4
	m	18	62.7	±7.1	18	64.1	±9.2	7	60.1	±9.7	7	60.5	±6.9
CMJ_P_max__100	f	9	48.6	±4.7	11	46.6	±4.9	12	45.2	±4.0	14	44.3	±4.6
	m	17	54.3	±6.1	18	54.9	±7.3	5	55.4	±9.1	7	53.3	±5.5
SJ_P_max__0	f	14	49.8	±6.6	13	47.8	±6.2	16	49.2	±4.1	16	47.6	±6.7
	m	18	57.3	±6.0	18	58.1	±8.8	7	57.2	±8.4	7	57.7	±5.5
SJ_P_max__100	f	9	46.2	±5.1	10	42.6	±6.1	12	42.5	±4.2	14	40.2	±4.2
	m	17	50.6	±6.2	18	51.2	±7.3	5	52.1	±7.8	7	48.9	±6.4

Descriptive data by sex for the non-injured (n_INJ) and the post-injured (p_INJ) groups are given for each measurement parameter with the number (n), mean, and standard deviation (SD). Maximal strength parameters (F_max_) are given in newtons per kilogram of body mass (N/kg) Explosive strength parameters (P_max_) for countermovement jump (CMJ) and squat jump (SJ) without (_0) or with an additional load of 100% body weight (_100) are given in watts per kilogram of body mass (W/kg).

**Table 5:** Statistical results for comparisons between sexes with non-injured and post-injured groups at T1 and T2.

Parameter	n_INJ						p_INJ					
	T1			T2			T1			T2		
	*p*		r	*p*		r	*p*		r	*p*		r
Body Mass	<0.001	***	0.89	<0.001	***	0.91	<0.001	***	0.97	<0.001	***	0.97
F_max__70	0.034	*	0.44	0.002	**	0.64	0.003	**	0.75	0.001	**	0.80
F_max__100	0.777		0.07	0.135		0.32	0.130		0.48	0.154		0.42
CMJ_P_max__0	<0.001	***	0.73	<0.001	***	0.81	0.089		0.46	0.004	**	0.75
CMJ_P_max__100	0.011	*	0.61	0.003	**	0.66	0.027	*	0.70	0.002	**	0.82
SJ_P_max__0	0.002	**	0.62	<0.001	***	0.72	0.015	*	0.64	0.003	**	0.77
SJ_P_max__100	0.133		0.37	0.004	**	0.66	0.037	*	0.67	0.007	**	0.71
SJ_Pmax_100	0.133		0.37	0.004	**	0.66	0.037	*	0.67	0.007	**	0.71

Statistical results of the sex-specific group comparison of non-injured (n_INJ) and post-injured (p_INJ) groups at T1 and T2 are presented for each measured parameter. The significant differences (* = *p* < 0.05, ** = *p* < 0.01, *** = *p* < 0.001) are highlighted and accompanied by the effect size.

## Data Availability

Due to the specificity of the data from the defined group of competitive athletes, the data presented in this study are available upon request from the corresponding author.

## Abbreviations

The following abbreviations are used in this manuscript:

ACL	Anterior cruciate ligament
CMJ	Countermovement jump
EC	European Cup
FIS	International Ski Federation
F_max_	Isometric maximal strength (force)
ISS	Injury surveillance system
MLD	Muscle diagnostic test procedure (Muskelleistungsdiagnostik)
N_INJ	Non-injured group
P_INJ	Post-injured group
P_max_	Explosive strength (mechanical peak power)
RTS	Return to sport
RTP	Return to performance
SJ	Squat jump
T1	Test 1
T2	Test 2
WC	World Cup
